# CIK Cells and HDAC Inhibitors in Multiple Myeloma

**DOI:** 10.3390/ijms18050945

**Published:** 2017-04-29

**Authors:** David Stephan, Hans Weiher, Ingo G.H. Schmidt-Wolf

**Affiliations:** 1 Department of Internal Medicine III, Center for Integrated Oncology (CIO), University Hospital Bonn, Rheinische Friedrich-Wilhelms-Universität Bonn, Sigmund-Freud-Straße 25, 53105 Bonn, Germany; david.stephan1701@gmail.com; 2 Hochschule Bonn-Rhein-Sieg, Von-Liebig-Straße 20, 53359 Rheinbach, Germany; hans.weiher@h-brs.de

**Keywords:** multiple myeloma, cytokine-induced killer cells, CIK cells, histone deacetylase inhibitors, immunotherapy, cancer treatment

## Abstract

Multiple myeloma is the second most common hematological malignancy. Despite all the progress made in treating multiple myeloma, it still remains an incurable disease. Patients are left with a median survival of 4–5 years. The combined treatment of multiple myeloma with histone deacetylase inhibitors and cytokine-induced killer cells provides a promising targeted treatment option for patients. This study investigated the impact of a combined treatment compared to treatment with histone deacetylase inhibitors. The experiments revealed that a treatment with histone deacetylase (HDAC) inhibitors could reduce cell viability to 59% for KMS 18 cell line and 46% for the U-266 cell line. The combined treatment led to a decrease of cell viability to 33% for KMS 18 and 27% for the U-266 cell line, thus showing a significantly better efficacy than the single treatment.

## 1. Introduction

Multiple myeloma (MM) is a hematological malignancy which is characterized by proliferation and subsequent clonal expansion of atypical plasma cells which produce an excess of monoclonal immunoglobulin [1,2]. MM cells lead to osteolytic bone destruction by inducing activity of osteoclasts while suppressing the activity of osteoblasts. Additional to the activation of pre-existing osteoclasts MM cells stimulate osteoclast neogenesis. On the other hand osteoclasts produce factors promoting tumor growth. The constant degradation of bone mass leads to lesions in the bone [3]. One of the reasons MM is so difficult to cure is that MM cells have a close relationship to the bone marrow micro-environment where the cells receive multiple signals maintaining their longevity and protection from drug-induced apoptosis [4]. Additionally, MM cells escape the immune system by reducing the number of natural killer (NK) cell inducing ligands, for example NKG2D, on the surface of the cell masking them from attacks by NK cells [5].

Cytokine-induced killer (CIK) cells are highly cytotoxic natural killer T (NKT) cells able to lyse tumor cells. They were shown to proliferate faster than lymphokine activated killer cells and have a higher cytotoxicity [6,7]. The cytotoxicity is not linked to major histocompatibility complex which differentiates them from normal cytotoxic T-lymphocytes. The high cytotoxicity derives from the subgroup of CD3+ and CD56+ co-expressing cells forming a new subset of NKT cells [8]. CIK cells have already been tested against a variety of different tumor types. Clinical studies showed that CIK cell treatment is well tolerated by patients and provided better results than conventional therapies. Additionally some studies suggest that CIK cells counteract viral infections [9].

NKG2D receptors are found on the cell surface of NK cells and NKT cells; they are categorized as homodimeric C-type lectin-like NK receptors. They bind to various structural homologues of MHC-I like MICA or MICB, which are up-regulated in stressed cells and epithelial tumors [10]. Therefore, NKG2D-ligand interactions can mediate anti-tumor and anti-viral immune responses related to low level signals [11]. After binding to a NK cell activating ligand the NKG2D receptor sends signals to the NK cell, through tyrosine-based activation molecules, which results in the up-regulation of their killing ability [12].

Histone deacetylase (HDAC) inhibitors are drugs that can prevent the deacetylation of histones binding to DNA. HDAC inhibitors bind directly to the site of deacetylation leaving an increased amount of deacetylated DNA because the active site for HDAC is blocked [13]. Thereby, HDAC inhibitors can induce cell cycle arrest, re-activate silenced tumor suppressor genes and even induce apoptosis [14]. Furthermore, HDACs also target non-histone proteins such as transcription factors and DNA repair enzymes, which can be activated by HDAC inhibitors [15]. HDAC inhibitors have already been proven effective and are used in the treatment of MM improving the outcome. In 2015 the FDA approved panobinostat which was successfully tested in combination with bortezomib leading to a prolonged mean survival rate in a placebo study [16]. The aim of this study was to test the combined use of HDAC inhibitors and CIK cells and to test whether this combination leads to a reduced cell viability of MM cells. The basis of this study is the observation that both HDAC inhibitors and CIK cells, on their own, lower the cell viability of tumor cells [17], however, have not been studied in combination yet.

## 2. Results

In first experiments, it was tested to what extend HDAC inhibitors kill CIK cells. In the next step, the most potent ratio of CIK to tumor cells was determined. The last step was testing the HDAC inhibitors Sodium Butyrate (SB), Valproic Acid (VPA) and Trichostatin A (TSA) in combination with CIK cells on the tumor cell lines, KMS-18 and U-266.

### 2.1. The Effect of HDAC Inhibitors on Cell Viability of CIK Cells

**Figure 1:**
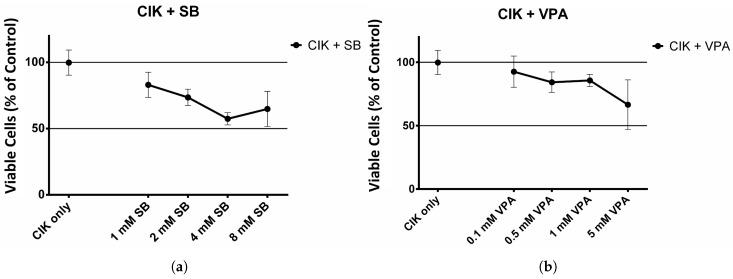

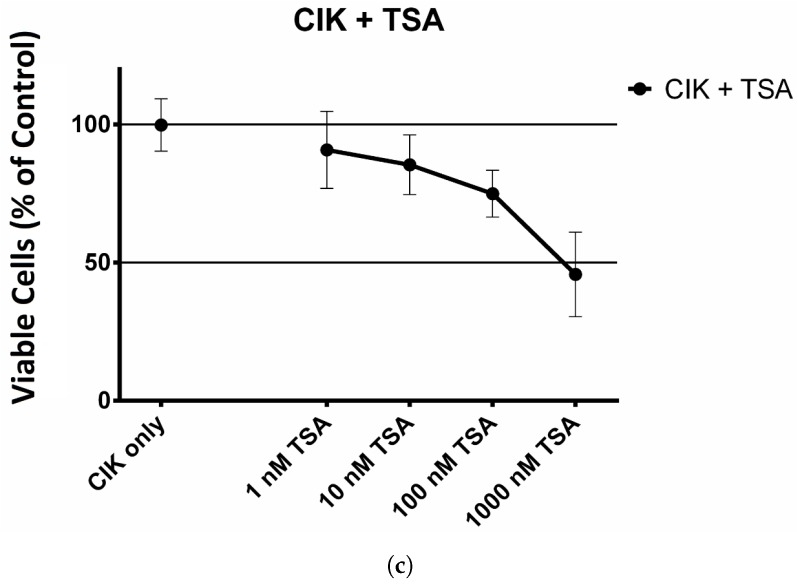
Effect of histone deacetylase (HDAC) inhibitors on cytokine-induced killer (CIK) cells. (**a**) Effect of Sodium Butyrate (SB) on CIK cells. CIK cells were incubated with H_2_O, 1, 2, 4, 8 mM SB for 24 h. The experiment was performed 6 times; (**b**) Effect of Valproic Acid (VPA) on CIK cells. CIK cells were incubated with H_2_O, 0.1, 0.5, 1, 5 mM VPA for 24 h. The experiment was performed 6 times; (**c**) Effect of Trichostatin A (TSA) on CIK cells. CIK cells were incubated with DMSO, 1, 10, 100, 1000 nM TSA for 24 h. The experiment was performed 6 times.

Figure 1a shows the cell viability of CIK cells in the presence of SB. Cell viability was calculated in comparison to the cell viability of the untreated CIK control. The viability decreases when the CIK cells are incubated with Sodium Butyrate.

Figure 1b shows the results for the HDAC inhibitor VPA. When incubated with VPA cell viability decreases.

Figure 1c shows the cell viability of CIK cells after incubation with TSA. Incubation with the HDAC inhibitor TSA leads to a significant decrease in cell viability.

### 2.2. Finding the Optimum Ratio of CIK Cells to Tumor Cells

**Figure 2:**
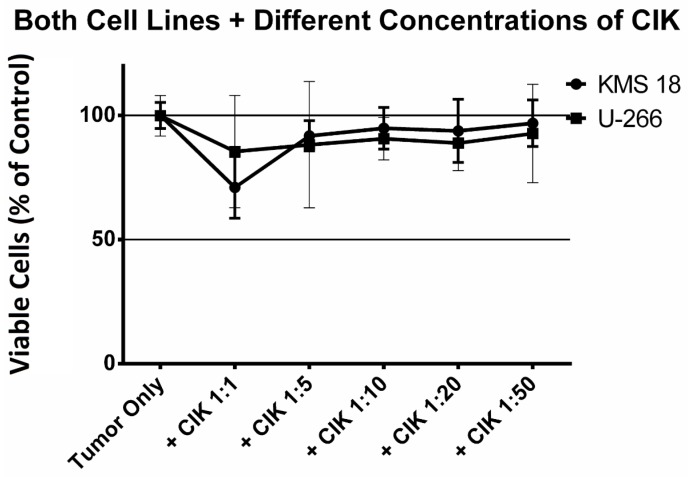
Effect of different ratios of CIK cells on both multiple myeloma cell lines. Tumor cells were incubated with ratios of 1:1, 1:5, 1:10, 1:20 and 1:50 tumor cells to CIK cells for 24 h. The experiment was performed 6 times for the tumor only and 1:1 ratio of KMS 18 and 9 times for the other KMS 18 related ratios. For the U-266 cell line the experiment was performed 3 times for each ratio.

After it was verified that CIK cells survive being exposed to the HDAC inhibitors, it had to be determined in which ratio the CIK cells should be co-cultured with the tumor cells in order to obtain the lowest possible cell viability. The results are shown in Figure 2. For KMS 18 cell line, the lowest cell viability was achieved with a ratio of 1:1. Ratios of 1:5, 1:10, 1:20 and 1:50 resulted in higher cell viabilities.

Looking at the U-266 cell line, the 1:1 ratio proved to be the most potent ratio. Therefore, all following experiments were performed with a 1:1 ratio of CIK to MM cells.

### 2.3. Effect of HDAC Inhibitors and CIK Cells on KMS 18 cells

**Figure 3:**
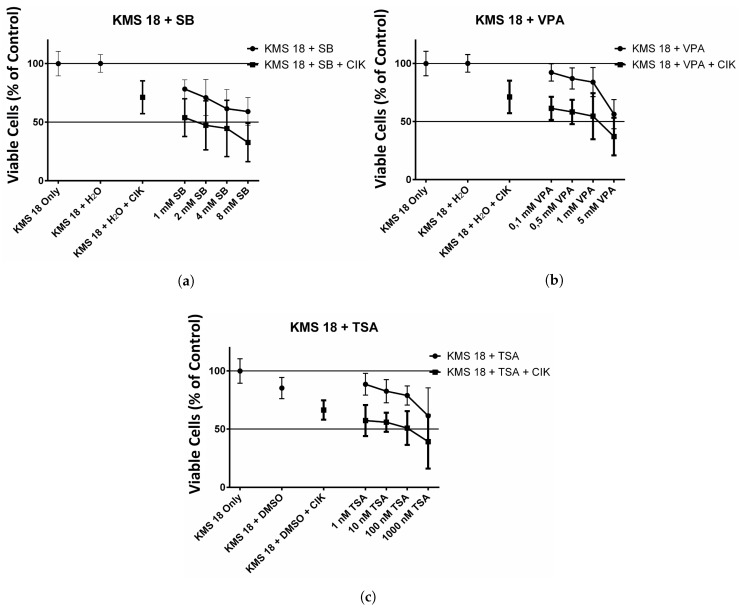
Effect of HDAC inhibitors on KMS 18 cell line with and without CIK cells. (**a**) Effect of SB on KMS 18 cells with and without CIK cells. The KMS 18 cells were incubated with 1, 2, 4, 8 mM SB for 24 h. Afterwards, CIK cells or new medium was added to the respective wells and incubated for another 24 h. The experiment was performed 9 times; (**b**) Effect of VPA on KMS 18 cells with and without CIK cells. The KMS 18 cells were incubated with 0.1, 0.5, 1, 5 mM VPA for 24 h. Afterwards, CIK cells or new medium was added to the respective wells and incubated for another 24 h. The experiment was performed 9 times; (**c**) Effect of TSA on KMS 18 cells with and without CIK cells. The KMS 18 cells were incubated with 1, 10, 100, 1000 nM TSA for 24 h. Afterwards, CIK cells or new medium was added to the respective wells and incubated for another 24 h. The experiment was performed 9 times.

Finally, tumor cells were incubated with HDAC inhibitors and CIK cells. Figure 3a shows the cell viability of KMS 18 cells in combination with SB in the presence and absence of CIK cells. The results show a clear negative trend in cell viability when cells were incubated with SB and even more with the combination of SB and CIK cells. The difference between the incubation with only the HDAC inhibitor and the HDAC inhibitor in combination with CIK cells was significant (* p= 0.0152).

Figure 3b shows a similar negative trend of cell viability for the incubation with VPA. The cell viability decreases with increasing dosage of VPA and again there was a significantly (* p= 0.0152) higher decrease of cell viability when MM cells were incubated together with VPA and CIK cells.

Figure 3c shows the cell viability of KMS 18 when incubated with TSA. The negative trend of cell viability was also present for the HDAC inhibitor TSA. A significantly (** p= 0.0043) bigger decrease could be observed when the combined treatment of HDAC inhibitor and CIK cells was used.

### 2.4. Effect of HDAC Inhibitors and CIK Cells on U-266 Cells

**Figure 4:**
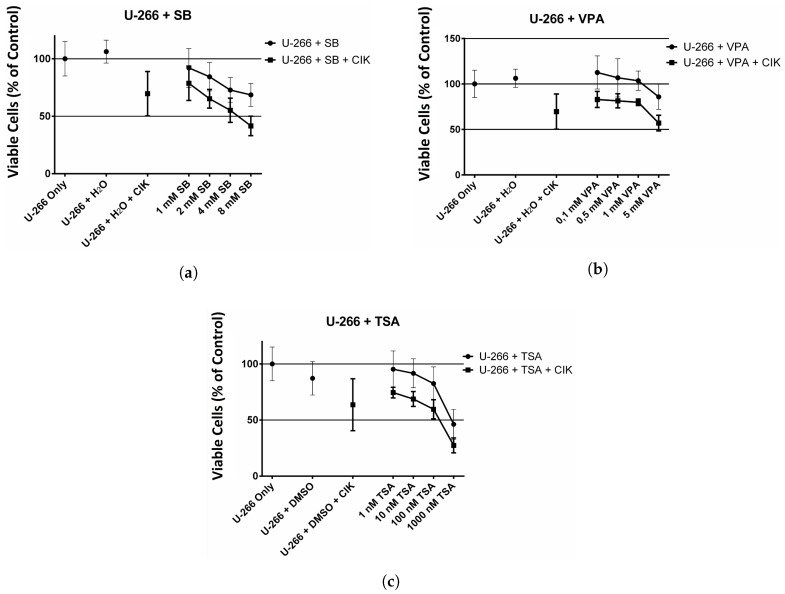
Effect of HDAC inhibitors on U-266 cell line with and without CIK cells. (**a**) Effect of SB on U-266 cells with and without CIK cells. U-266 cells were incubated with 1, 2, 4, 8 mM SB for 24 h. Afterwards, CIK cells or new medium was added to the respective wells and incubated for another 24 h. The experiment was performed 9 times; (**b**) Effect of VPA on U-266 cells with and without CIK cells. U-266 cells were incubated with 0.1, 0.5, 1, 5 mM VPA for 24 h. Afterwards, CIK cells or new medium was added to the respective wells and incubated for another 24 h. The experiment was performed 9 times; (**c**) Effect of TSA on U-266 cells with and without CIK cells. U-266 cells were incubated with 1, 10, 100, 1000 nM TSA for 24 h. Afterwards, CIK cells or new medium was added to the respective wells and incubated for another 24 h. The experiment was performed 9 times.

Analogous experiments were performed with the U-266 cell line. The results of the SB treatment are visualized in Figure 4a. As previously seen for the KMS 18 cell line, there is a negative trend in cell viability of U-266 cells when incubated with SB. The decrease of cell viability is significantly (* p= 0.026) higher when SB is combined with CIK cells in contrast to the single treatment with SB alone.

Figure 4b shows cell viability of U-266 cells after the treatment with VPA. Similar to the treatment with HDAC inhibitor SB the treatment with VPA showed a decrease of cell viability. And again the decrease of cell viability is significantly (** p=0.0022) higher if HDAC inhibitor and CIK cells were used in combination. Towards the higher concentrations of VPA cell viability decreases more strongly.

Figure 4c shows the results after treatment of U-266 cell with TSA. Since TSA was dissolved in DMSO the initial cell viability was lower than for the previous HDAC inhibitors. Again, an increase of concentration of HDAC inhibitor led to decreased cell viability. The combination of TSA and CIK cells led to a significantly (* p=0.026) higher decrease in cell viability.

## 3. Discussion

Tumor cells have shown an up-regulation of NK-cell activating ligands after treatment with some HDAC inhibitors [18]. Here HDAC inhibitors were tested hoping that an up-regulation of NK-cell activating ligands would lead to an increased recognition and subsequent lysis of MM cells. This was done in vitro with two multiple myeloma cell lines, KMS 18 and U-266. The experiment showed that a combination of CIK cells and HDAC inhibitors significantly reduce cell viability of MM cells in vitro. Additionally, we saw that CIK cells survive exposure to HDAC inhibitors and therefore can be used in a combined treatment of MM. However, no flow cytometry was performed, therefore it was not possible to determine if up-regulation of NK-cell activating ligands took place.

CIK cells have already been proven to be effective in the treatment of various tumors and were found to be very effective in increasing the quality of life in cancer patients as well as in improving the immune system especially in patients suffering from late stage malignant tumors [19]. CIK cells have also been proven to be effective in other combinatory treatments for example when used in the treatment of patients receiving chemotherapy. Here the treatment was found to prolong the survival of patients. It also enhanced the immune functions of the patients and eased the complications originating from chemotherapy [20]. Since CIK cells are derived from the patients blood they are well tolerated by the patient. The combinatory treatment of MM by HDAC inhibitors and CIK cells may constitute a new treatment method in the future based on the increased recognition of MM cells by CIK cells and to an improved immune function.

For SB it has been established by flow cytometry that it leads to an up-regulation of MICA [21], whereas for the other two HDAC inhibitors such studies have yet to be conducted. In the recent past many NK cell activating substances relying on surface ligands have been approved for treatment of tumors. AFM13 is a bi-specific antibody that has been reported to lead to a significant increase in NK cell activation [22]. Elotuzumab is a monoclonal antibody specifically designed to target SLAMF7 in MM. It showed a high efficacy when combined with proteasome inhibitors like bortezomib [23]. Another human monoclonal antibody targeting MM cell surface proteins is Daratumumab targeting CD38 which is over-expressed on MM cells. Daratumumab was found to be well tolerated by patients and led to cell death of MM cells via several mechanisms [24]. A study combining Daratumumab with proteasome inhibitors and dexamethasone showed promising results for patients suffering from relapsed and refractory MM leading to a significantly prolonged progression-free survival [25]. If all three HDAC inhibitors up-regulate NK-cell activating ligands a combination of CIK cells HDAC inhibitors and monoclonal antibodies might result in stronger antibody labeling and an even greater cytotoxic activity of the CIK cells and NK-cells and lead to prolonged survival rate of patients.

## 4. Materials and Methods

### 4.1. Cells

KMS 18 (University of Leeds, Leeds, England) and U-266 (DSMZ, Braunschweig, Germany) are both cell lines derived from patients suffering from multiple myeloma. Both cell lines were cultured in 75 cm${}^{2}$ culture flasks using RPMI-medium (PAN Biotech, Aidenbach, Germany) supplemented with 10% fetal calf serum (FCS) (Gibco Life Technologies, Darmstadt, Germany) and 1% penicillin/streptomycin (P/S) (Life Technologies, Darmstadt, Germany). Cells were kept at 37 °C and 5% CO_2_. New Medium was added every 3 days and completely replaced at least once a week or whenever deemed necessary. Cells were split weekly or whenever splitting was required.

CIK cells were cultured in RPMI-medium supplemented with 10% FCS and 1% P/S. Additionally the medium was buffered with 2.5% HEPES buffer (PAN Biotech). Every 3 days part of the medium was renewed and 300 U·mL${}^{-1}$ interleukin-2 (IL-2) (Immuno Tools, Friesoythe, Germany, and, Novartis Pharma GMBH, Nuernberg, Germany) was added to the culture flask. CIK cells were not centrifuged, unless part of the culture was harvested for further experiments.

### 4.2. Buffy Coat

In a sterile glass container phosphate buffered saline (PBS) and 1% bovine serum albumin (BSA) (Life Technologies; PAA, Coelbe, Germany) were mixed with 50 mL of blood. Afterwards, a Falcon tube containing 15 mL Pancoll (PAN Biotech) was carefully overlaid with the blood PBS/BSA mixture. The tube was centrifuged at 1000 rpm for 30 min while the centrifuge break was switched off and the centrifuge was allowed to slowly come to a stop.

After centrifugation the whitish middle layer containing the lymphocytes was carefully taken off with a pipette and transferred into a new falcon tube. The tube was filled to 50 mL and centrifuged for 7 min at 800 rpm for this the break on the centrifuged was switched back on. Lymphocytes were washed three times with 50 mL PBS. After the first washing step re-suspension of the pellet was done in 10 mL erylyse buffer and the tube was incubated on ice for 10 min. In the second washing step a small amount of resuspended cells was taken for counting in a hemocytometer.

After washing the cell number was adjusted to to 5×10${}^{6}$ mL${}^{-1}$ and the cell suspension was split up so that there was a maximum of 30 mL in one incubation flask. The flask(s) were incubated at 37 °C and 5% CO_2_ in order to allow the dendritic cells to settle on the ground and attach to the bottom of the flask. Afterwards, the medium containing the peripheral lymphocytes was transferred to a new flask which was filled up to 40 mL with CIK cell medium. In the end 1000 U·mL${}^{-1}$ interferon gamma (IFN-γ) were added to the cell.

The next day the cells once had to be supplied with:

- 300 U·mL${}^{-1}$ IL-2
- 50 ng·mL${}^{-1}$α-CD3
- 100 U·mL${}^{-1}$ interleukin1 β (IL-1 β)

Additionally, CIK cells have to be supplemented with 300 U·mL${}^{-1}$ IL-2 every three days.

### 4.3. MTT Assay

In order to measure and compare the cytotoxicity of the CIK cells a 3-(4,5-dimethylthiazol-2-yl)-2, 5-diphenyltetrazolium bromide (MTT) assay was performed. The MTT assay was done using 96 well plates. In each well 1×10${}^{4}$ MM cells were plated out with 50 μL of medium and one HDAC inhibitor.

The HDAC inhibitors were used at the following concentrations:
  Sodium Butyrate:
8 mM4 mM2 mM1 mM  Valproic Acid:
5 mM1 mM0.5 mM0.1 mM  Trichostatin A:
1 mM100 nM10 nM1 nM

96-well plates were incubated for 24 h. After incubation 50 μL of CIK cells were pipetted into the wells. For control 50 μL pure medium was added. On the last day 80 μL medium was pipetted out of the wells, after centrifugation of the plate, and 55 μL of MTT reagent was added into the wells. The plates were incubated for 45 min. After a second centrifugation 55 μL were removed from the wells and 80 μL DMSO was pipetted in the wells. Then the plates were put on a shaker at 37 °C and 350 rpm for 10 min after which the absorbance of each well was measured and documented in a spectrophotometer at 560 nm. In this case, a Glomax Multi Detection System (Promega, Madison, WI, USA) was used.

Cell viability was calculated from the values gathered with the photometer.

### 4.4. Statistical Analysis

Statistical analysis was performed using the program Graph Pad Prism 5 (Graph Pad Software Inc., La Jolla, CA, USA). Data were presented as mean with standard deviation. In order to determine statistical significance Student’s t-test was used. The statistical significance limit was set to *p* < 0.05 (ns = not significant, * *p* < 0.05, ** *p* < 0.01, *** *p* < 0.001).

## Abbreviations

MM	Multiple myeloma
NK	natural killer
CIK	Cytokine-induced killer
NKT	natural killer T
HDAC	Histone deacetylase
SB	Sodium Butyrate
VPA	Valproic Acid
TSA	Trichostatin A
FCS	fetal calf serum
P/S	penicillin/streptomycin
IL-2	interleukin-2
PBS	phosphate buffered saline
BSA	bovine serum albumin
IFN-γ	interferon gamma
IL−1β	interleukin−1β
MTT	3-(4,5-dimethylthiazol-2-yl)-2,5-diphenyltetrazolium bromide
